# Platelet-Rich Plasma Favors Proliferation of Canine Adipose-Derived Mesenchymal Stem Cells in Methacrylate-Endcapped Caprolactone Porous Scaffold Niches

**DOI:** 10.3390/jfb3030556

**Published:** 2012-08-09

**Authors:** Francisco Javier Rodríguez-Jiménez, Teresa Valdes-Sánchez, José M. Carrillo, Mónica Rubio, Manuel Monleon-Prades, Dunia Mercedes García-Cruz, Montserrat García, Ramón Cugat, Victoria Moreno-Manzano

**Affiliations:** 1 Neuronal Regeneration Lab, Centro de Investigación Principe Felipe, València 46012, Spain; Email: frodriguez@cipf.es (F.J.R.); teresavalsan@gmail.com (T.V.); 2 Medicine and Surgery Department, CEU-Cardenal Herrera University, Moncada 46115, Spain; Email: jcarrill@uch.ceu.es (J.M.C.); mrubio@uch.ceu.es (M.R.); 3 Fundación García Cugat, Barcelona 08006, Spain; Email: montse.garcia@sportrauma.com (M.G.); secretaria@sportrauma.com (R.C.); 4 Centre for Biomaterials and Tissue Enginering, Universitat Politècnica de València, València E-46022, Spain; Email: mmonleon@ter.upv.es (M.M.); dugarcru@ter.upv.es (D.M.G.); 5 Artroscopia GC, Hospital Quirón, Barcelona 08023, Spain

**Keywords:** adipose tissue, mesenchymal stem cells, PRGF, caprolactone, scaffold

## Abstract

Osteoarticular pathologies very often require an implementation therapy to favor regeneration processes of bone, cartilage and/or tendons. Clinical approaches performed on osteoarticular complications in dogs constitute an ideal model for human clinical translational applications. The adipose-derived mesenchymal stem cells (ASCs) have already been used to accelerate and facilitate the regenerative process. ASCs can be maintained *in vitro* and they can be differentiated to osteocytes or chondrocytes offering a good tool for cell replacement therapies in human and veterinary medicine. Although ACSs can be easily obtained from adipose tissue, the amplification process is usually performed by a time consuming process of successive passages. In this work, we use canine ASCs obtained by using a Bioreactor device under GMP cell culture conditions that produces a minimum of 30 million cells within 2 weeks. This method provides a rapid and aseptic method for production of sufficient stem cells with potential further use in clinical applications. We show that plasma rich in growth factors (PRGF) treatment positively contributes to viability and proliferation of canine ASCs into caprolactone 2-(methacryloyloxy) ethyl ester (CLMA) scaffolds. This biomaterial does not need additional modifications for cASCs attachment and proliferation. Here we propose a framework based on a combination of approaches that may contribute to increase the therapeutical capability of stem cells by the use of PRGF and compatible biomaterials for bone and connective tissue regeneration.

## 1. Introduction

In the field of regenerative medicine, an increasing number of strategies propose a combination of different therapeutic approaches that have improved the regeneration process when applied alone. Numerous strategies of tissue engineering presently under development in humans and animals include transplants of stem cells to regenerate damaged tissues and organs and constitute a promising approach for bone and adjacent tissues regeneration [1]. The use of mesenchymal stem cells to repair bones and joint tissues, such as cartilage and tendon, offers therapeutic alternatives [2,3] and constitute a real hope for the use in traumatic, degenerative and inflammatory disorders associated with these tissues [4,5]. Adipose-derived stem cells (ASCs) constitute a source of stem cells of easy and rapid isolation from adipose tissue that can differentiate to multiple lineages, including osteoblast and chondrocytes [6,7,8]. Non-aggressive invasive biopsy from subcutaneous adipose tissue and suction-assisted lipectomy (liposuction) are frequent techniques used to obtain adult adipose-derived stem cells. New approaches for ASC amplification and isolation are being developed [8,9]. Their main goal is to generate sufficient number of stem cell for clinical applications, including autologous transplantation approaches that would avoid undesired immunoreactions. In fact, during last decade, much effort is being implemented to increase the yield, efficiency and therapeutical capability of stem cells, including the use of different growth factors as those associated with platelet rich plasma growth factors (PRGF) [10]. PRGF might constitute an autologous source of growth factors (GFs) that can contribute to tissue regeneration [11,12]. In fact, PRGF contains GFs that induce growth of mesenchymal stem cells (MSCs) and osteogenic lineage cells which can accelerate bone repair [13]. Therefore, a combination of ASCs and PRGF can produce a synergistic effect to increase the yield on bone formation and consolidation [14]. Additional strategies in tissue engineering in osteoarticular pathologies involve a combination of ASCs with coral scaffolds resulting in the successful repair of canine bone defects [1]. Moreover, a variety of polymeric scaffolds for replacement into the injured tissue has been of interest for several research groups during the last decade. In fact, the scaffold biocompatibility within the tissue environment has already been for different materials (reviewed in [15,16]). A variety of naturally derived and synthetic biomaterial scaffolds have been investigated as 3D environments for supporting stem cell growth. Natural biomaterials are often composed of elements found in the extracellular matrix that favor cell adhesion that improve cell culture and usually show biodegradability. Synthetic scaffolds can be synthesized to have a greater range of mechanical and chemical properties often offer greater reproducibility are easy to manipulate and have no risk of viral infection. Additionally, they can be reproducibly manufactured on a large scale, with a variety of specific properties. Despite good biocompatibility of biomaterials, many synthetic scaffolds lack cell-adhesive properties and need additional modifications to allow cell-surface interactions. Caprolactone 2-(methacryloyloxy) ethyl ester (CLMA), allows enhanced cellular adhesion and proliferation [17]. CLMA generates scaffolds with controlled porosity for tissue engineering [18] that may favor the integration of stem cells in the damaged tissue that after differentiation would allow sites for regeneration in osteoarticular –related pathologies. ASCs isolated under aseptic conditions and amplified in GMP clinical-compatible criteria allow for better translational applications. Here we propose to create a biological and synergistic framework formed by a combination of PRGF, that positively contribute to the survival of cASCs, and the use of biomaterial scaffolds of CLMA as a compatible platform to fill the gap in the damaged tissue for optimal recovery. 

## 2. Results and Discussion

### 2.1. Canine ASC Isolation and Characterization

**Figure 1:**
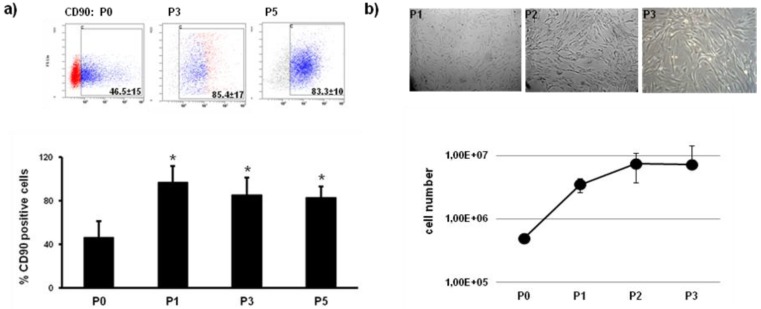
Canine adipose-derived mesenchymal stem cells (cASC). (**a**) Flow cytometry analysis of CD90 positive population of ASC at passages 0 (before adherent cell culture conditions), and passages 3 and 5 (in adherent conditions). * P < 0.05; (**b**) Upper panels: phase contrast microscopy representative images of ASC grown in adherent conditions at P1-3. Lower panel: Cell growth curve of ASC quantified from five independent samples at P1-3 and values were expressed as mean ± S.D. * P < 0.05.

Tissue engineering has become a promising strategy to overcome the limitations of autologous transplants for therapeutical applications and has grown exponentially in the last two decades. The usual approaches propose to transplant active elements such as cells for gene-therapy, stem cells or proteins within a porous degradable material known as a scaffold. Since stem cells are naturally engrafted in active 3D microenvironments *in vivo*, current tissue engineering approaches often try to mimic the stem cells niche. Many proposed methods for engineering replacement tissues accurately mimic these microenvironments by culturing stem cells in 3D biomaterial scaffolds. In general, 3D scaffolds provide an appropriate establishment of cell polarity in the environment and possess biochemical and mechanical properties similar to the damaged tissue. Here we obtained cASC under aseptic conditions to be amplified following GMP criteria compatible for clinical use. cASCs were obtained from five different dogs after inguinal fat biopsy. Twenty grams of subcutaneous fat were processed enzymatically (collagenase) washed and centrifuged several times until obtaining a cell concentrate in sterilized conditions. cASC enrichment and expansion, containing autologous serum in the growth medium, was achieved by the use of a Bioreactor with temperature, oxygen and CO_2_ controlled (Soluciones Bioregenerativas-Proteal, Barcelona, Spain). A minimum of 30 million cells are delivered within 2 weeks providing a rapid and aseptic method for stem cells production and further use in clinical applications. Two weeks after fat biopsy the concentrated cell suspension was further characterized by using CD90 as a stem cell marker. Flow cytometry analysis showed that half of the population at passage 0 positively reacts to CD90 hybridization (Figure 1a). CD34 (hematopoietic cell marker) hybridized with almost 40% of the remaining cell population (data not shown). Subsequently passages of cASCs in adherent conditions significantly enriched the CD90 positive population up to P5 (Figure 1a). Morphological analysis of cASC after three consecutive passages showed feature correlation and resemblance to mesenchymal stem cells (Figure 1b, upper panels). cASCs obtained from five different dogs were seeded (5 × 10^5 ^cells) and counted after three consecutive passages. A consistent and exponentially growing proliferative curve was found for all tested samples in growth medium containing 10% fetal bovine serum (Figure 1b). 

### 2.2. Influence of PRGF in cASC Growth

**Figure 2:**
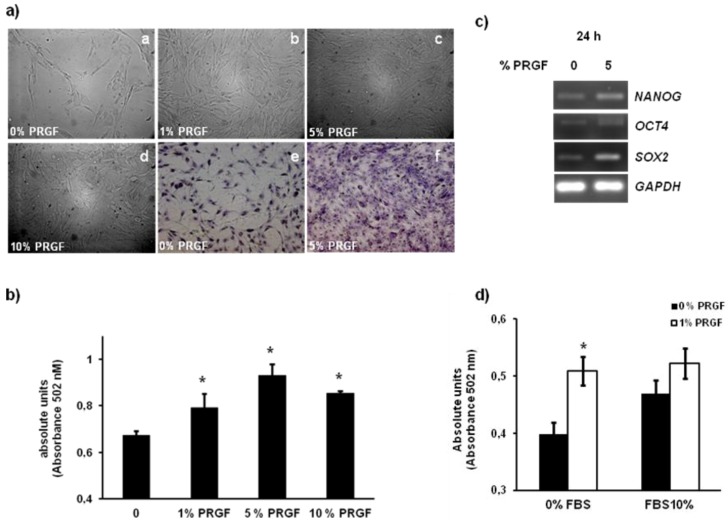
Plasma rich in growth factors (PRGF) treatment induce ASC proliferation. (**a**) Phase contrast microscopy images of cASC evidence an increased cell density in the presence of PRGF (1%, 5% or 10%) in a PRGF dose dependent manner (**a**–**e**; non-fixed and non-stained cells, **e**–**f**: fixed and stained with Giemsa); (**b**) Quantification of the proliferative activity of cASC in the presence or absence of PRGF (0, 1, 5 or 10%) for 24 hours, * P < 0.05 *vs*. 0% PRGF. The mean ± S.D. of three different experiments is represented; (**c**) Semiquantitative PCR stemness-related gene expression in cASC treated or not with PRGF (5%); (**d**) Quantification of the proliferative activity of cASC in the presence or absence of 1% PRGF for 24 hours, with or without FBS (10%) in the growth medium. * P < 0.05 *vs*. 0% PRGF. The mean ± S.D. of three different experiments is represented.

The use of PRGF constitutes a source of cell signaling molecules and can contribute to tissue regeneration [11,12], also in combination with stem cells to reduce the time of consolidation in distraction osteogenesis [19]. Since PRGF represents a source of growth factors, it can favor the adhesion and proliferation of mesenchymal stem cells on 3D ceramic scaffolds [20]. However, the benefits of PRGF are still under evaluation since inconsistent results have been reported. This controversy may be caused by individual variation in growth factor concentration in PRGF [21]. We determined the effects of increasing concentrations (1%, 5% and 10%) of PRGF in cASCs proliferation. Confluent cultures were reached when cASCs were treated with 5% PRGF for 24 hours (Figure 2a). Untreated cells and those treated with PRGF (5%) were stained with Giemsa to improve visualization (Figure 2a). Significantly increased proliferation (*P* ≤ 0.05) of cASCs after treatment with 1%, 5% and 10% PRGF was quantified by MTS assay (Promega, USA) (Figure 2b). Transcriptional expression of the pluripotent markers *Nanog*, *Sox2* and *Oct4* was evaluated by semi-quantitative PCR. We detected higher presence of transcripts for all three pluripotent markers when cASCs where treated with 5% PRGF for 24 hours that suggests a higher self-renewal of cASCs (Figure 2c). A cultured supplement composed of 3% human platelet-poor plasma (hPPP) with a cytokine cocktail formed by epidermal growth factor (EGF), basic fibroblast growth factor (bFGF) and platelet-derived growth factor-bb (PDGFbb) improves ASC proliferation more than FBS. The addition of hPRGF to the described supplement or hPRGF alone also increases ASC expansion [10]. In line with these results, we observed that treatment of cASCs with a combination of FBS with PRGF does not change cell viability rates in comparison to those obtained with PRGF alone (Figure 2d). Therefore, our results suggest that growth factors contained in PRGF might be sufficient to improve the yield of cASCs.

### 2.3. cASCs Exhibit Improved Adaptation to CMLA Scaffolds and Higher Proliferation When Treated with PRGF

To our knowledge, there is a lack of proposals that combine stem cells, scaffolds and the PRGF, an optimal autologous source of growth factors. For instance, dental implants, using tissue engineered bone with natural fibrin scaffolds, stem cells and PRGF alone or in combination was previously proposed in dog [22]. The authors show a significant increase of bone-implant contact in dogs treated with a combination of mesenchymal stem cells, PRGF and fibrin. Fibrin has many advantages as a cell delivery vehicle in terms of biocompatibility, biodegradation and hemostasis [23,24] and can provide a permissive environment for cell growth with increased proliferation and survival of human bone marrow stromal cells in porous scaffolds [25]. This bioactive factor/scaffold has disadvantages when used as scaffolds, such as low rigidity that can produce shrinkage of scaffols and rapid degradation that would not permit proper bone regeneration. Therefore, other proposals suggest the use of fibrin as an adjunt element for sustaining osteogenic capacity of stem cells in mineralized scaffolds [26]. PRGF has also been considered as a natural scaffold [19] with advantages such as flexibility, non toxic, no immune reaction, complete resorption [27,28]. However, the use of PRGF as a gel can present similar disadvantages to fibrin. 

A more consistent material such as those formed by synthetic materials might be required for bone regeneration. Pieri and colleagues propose the use of mesenchymal stem cells, platelet-rich plasma and fluorohydroxyapatite (FH) scaffold [29]. In general, FH is a main component of bone mineral and accepted as a bioactive material with biocompatibility with hard tissues [30], despite its low degradation rate, mechanical strength and osteoinductive potential [7,8]. Autologous mesenchymal stem cells loaded onto porous ceramic cylinders of hydroxyaparite (65%) and p-tricalcium phosphate ceramic (35%) promoted faster consolidation of the fracture [31]. No additional growth factor enrichment was performed in this work and the observed high fold increase in the number of mesenchymal stem cells was explained through the culture expansion process. However, this is a time consuming process and porous ceramic presents a load-bearing capacity for stem cells and tends to fracture affected bones in dogs. Adequate cell adhesion to biomaterial implants is critical for a proper cell supply into the hosted tissue. Although porous scaffolds for bone tissue engineering present good biocompatibility and can be integrated with biological cells or molecules to regenerate tissues [32], some synthetic biomaterials need a previous additional modification to allow cell-surface interactions and improve adhesion properties of cells. 

**Figure 3:**
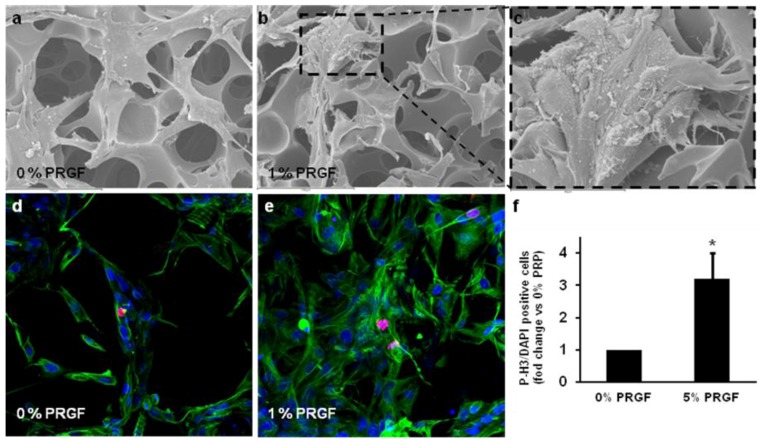
PRGF treatment induces cASC proliferation in a methacrylate-endcapped caprolactone (CLMA) porously network. (**a**–**c**) Ultrastructural view of cASC seed onto CLMA scaffolds by scanning electron microscope; (**d**–**e**). Confocal images show immunolocalization of vimentin (stem-cell marker, green), P-Histone H3 (mitotic marker, red) and DAPI (nuclear marker, blue) in cASC-CLMA scaffolds treated or not with PRGF; (**f**) Quantification of P-Histone H3 positive cASC-CLMA cells treated or not with PRGF. At least 6 different fields of 3 different experiments were quantified and the mean ± S.D. is represented. *P < 0.05 *vs.* 0% PRGF.

Caprolactone 2-(methacryloyloxy) ethyl ester (CLMA), has previously allowed enhanced cellular adhesion and proliferation [17]. CLMA generates scaffolds with controlled porosity for tissue engineering [18] that may favor the integration of stem cells in the damaged tissue that would allow bone regeneration after differentiation. In fact, cASCs are able to adhere to CLMA (Figure 3a–c) without additional modifications of CMLA. cASCs cultured in CMLA were distributed throughout the scaffold and showed positive proliferative activity when treated with PRGF (Figure 3d–f).

cASCs seeded on CMLA and treated with 1% PRGF showed higher yield in comparison to cells cultured on scaffolds without PRGF (Figure 3d,e). Cell proliferation was quantified by counting Phospho-Histone H3 positive cells (mitotic marker) by immunocytochemistry. There were significantly more phospho-histone H3-positive cells in cultures treated with 1% PRGF (Figure 3f).

## 3. Experimental Section

### 3.1. Adipose Tissue Processing and Derived-mesenquimal Stem Cell Culture

A biopsy of 20 g of inguinal subcutaneous fat from five owner dogs with EPA (Early Psoriatic Arthritis) and 120 ml of blood were isolated in sterilized conditions and processed by using the kit DogStem^®^ distributed by Soluciones Bioregenerativas-Proteal (Barcelona, Spain) following its manufacturer instructions. Immediately after sample collection, fat biopsy and blood (in anti-coagulant container) were sent at 4 °C for cell isolation and amplification in GMP conditions. Two weeks after biopsy, we analyzed the isolated and concentrated cells. The cells were maintained in growth medium: DMEM (**Life Technologies S.A.**, Alcobendas, Spain) supplemented with 10% heat-inactivated fetal bovine serum (FBS), 2 mM L-glutamine, 100 units/mL penicillin and 100 µg/mL streptomycin at 37 °C, 20% O_2_ and 5% CO_2._ All cell analysis was performed up to passage 8. All the sample collection was approved and certified by the dog owners with an informed consent.

### 3.2. FACS Analysis

ASC suspension was assayed for cell surface protein expression by flow cytometry (FC500, Cultek, Madrid, Spain). Cells at passage 0, 1, 3 and 5 were pelleted, resuspended in PBS at a concentration of 10^5^ cells/µL, and stained with 1:50 dilution of CD90 antibody conjugated with PE (BD Pharmigen, New Jersey, USA). Cells were incubated in the dark for 45 min at room temperature and then washed three times with PBS and resuspended in 0.3 mL of cold PBS for FACS analysis. The mean ± S.D. of the 5 different tested samples were determined. 

### 3.3. PRGF Preparation and ASC Viability and Proliferation

The PRGF, isolated from three different dogs, were prepared following the standardized method previously described by Anitua and collegues [33]. The cell viability and proliferative activity was determined by The CellTiter 96^®^ AQueous Non-Radioactive Cell Proliferation Assay (Promega Co., Madison, WI, USA) following the manufacturer´s instructions. Briefly, 10^5^ ASC at passages 2–5 were seeded onto 96 well plates and were allowed to grow for 24 hours in growth medium. After removing the growth medium the cells were treated with different concentrations of PRGF: 0%, 1%, 5% or 10% in the presence or absence of 10% FBS and 2 Units/ml of heparin for 24 hours. Every condition was assayed in quadruplicate in three different experiments. The viability of cells at each assayed condition was expressed as the percentage ratio of the mean ± S.D. of colorimetric signal from treated cells in the presence of PRGF in comparison with cells in the absence of PRGF.

The proliferative curves were assayed in growth medium (in the presence of 10% FBS). ASCs (5 × 10^5^) were seeded in 100 cm^2^ petri dishes and every third day trypan blue exclusion cell counting in a neubauer^®^ chamber was performed up to passage 5. The mean ± S.D. of absolute numbers of viable cells of the 5 different samples was determined. 

### 3.4. RNA Isolation and Semiquantitative RT-PCR

Total RNA was extracted by using the RNeasy Mini-kit (Qiagen, Germany) according to the manufacturer’s instructions. One µg of total RNA was reverse-transcribed in a total reaction volume of 50 µL at 42 °C for 30 min using random hexamer primers. The semi-quantitative PCR amplification was performed on a thermal cycler (Eppendorf, Germany) by using the following program, 3 min of denaturation at 95 °C followed by 30 cycles of 30 seconds at 95 °C, 15 seconds at 60 °C, 60 second at 72 °C and a final extension step at 72 °C for 4 minutes. The following primer sequences were used sequences: Sox2-Fw_5' AGTCTCCAAGCGACGAAA AA; Sox2-Rv_5': GCAAGAAGCCTCTCC TTGAA; Nanog-Fw_5': GAATAACCCGAATTGGAGCAG; Nanog-Rv_5': AGC GAT TCC TCT TCA CAG TTG; Oct4-Fw_5': GAGTGAGAGGCAACCTGGAG; Oct4-Rv_5´: GTGAAGTGAGGG CTCCCATA;GAPDH-Fw_5':CCATCTTCCAGGAGCGAGAT; GAPDH-Rv_5': TTCTCCATGGTG GTGAAGAC. The target gene value was normalized to the expression of the endogenous reference (GAPDH). A negative (without a prior reverse transcription reaction) control was always included. After amplification, 25 µL of each PCR mix was electrophoresed through a 2% (w/v) agarose gel with ethidium bromide (0.1 µg/mL) and visualized under an ultraviolet trans-illuminator (BioRad). The specific bands were analyzed by densitometry and the ratio with GAPDH expression was determined (all PCR amplifications were performed from 4 different experiments). 

### 3.5. Giemsa Staining

ASCs were fixed in 4% paraformaldehyde at room temperature for 10 min and washed with PBS before incubating in Giemsa solution (Sigma, St. Louis, MO, USA) for 30 min. The excess stain was removed by subsequent washes with distilled water. The cells were allowed to air dry and then visualized under the microscope. 

### 3.6. CLMA Scaffolds Preparation

Poly(methyl methacrylate) (PMMA) microspheres with a diameterof 90 ± 10 mm (Colacryl dp 300) were used as porogens by introducing them between two plates (of a self-built device whose distance could be controlled) and heating at 180 °C for 30 minutes to obtain the first template. This template shows the highest porosity attainable (that will yield the lowest porosity of the scaffold) with classical compaction values of 60%–65% for random mono-sized spherical particles. To obtain scaffolds with controlled porosity, the thickness of the obtained disk was first measured; then the disk was replaced in the mould and compressed at 180 °C for 30 min. The degree of compression was quantified by measuring the reduction in thickness. After cooling the template at room temperature, a mixture of caprolactone 2-(methacryloyloxy) ethyl ester (CLMA, as monomer), benzoin (1 wt %, as initiator) and ethylene glycol dimethacrylate (1 wt %, EGDMA) was introduced in the empty space between the PMMA spheres. The polymerization was carried out up to limiting conversion under a UV radiation source at room temperature. After polymerization took place, the porogen template was removed by Soxhlet extraction with acetone for 48 hours. The porous sample was maintained in Soxhlet with ethanol in order to extract low molecular weight substances for an additional 24 hours. Samples were dried in a vacuum to constant weight before characterization.

### 3.7. Morphological Characterization of CLMA Scaffolds

Morphological analysis of CLMA scaffolds were examined in a scanning electron microscope Jeol JSM-5410, SEM. All specimens were coated with a conductive layer of sputtered gold. The micrographs were taken at an accelerating voltage of 20 kV in order to ensure a suitable image resolution. When cells were seeded onto CLMA scaffolds before SEM analysis a previous fixation with 2.5% PFA plus 2% glutaraldehyde for 10 min at RT was performed and then dehydrated in graded ethanol concentrations. Critical point dryer (CPD) was performed on an Autosambri 814 instruments (Rockville, MD, USA) followed by sputter coated with gold before observation. 

### 3.8. Immunocytochemistry

Single cells (5 × 10^5^) were distributed in 2 µL per 2 mm^2^ CLMA scaffold (previously re-hydrated by overnight incubation in cell culture medium at 37 °C) or alone (without scaffold) individually set into 96 well plates, and incubated for 24 hours. Then ASCs were treated with 0 or 5% PRGF for an additional 24 hours in growth medium in the absence of FBS and with 2 Units/ml of heparin. Subsequently, the cells were fixed with 4% paraformaldehyde at room temperature for 10 min and washed with PBS, permeabilized with a PBS solution containing 0.1% Triton X-100 and blocked with 5% goat serum in PBS for 1 h. The following primary antibodies were diluted in blocking solution and incubated overnight at 4 °C. Vimentin (α-mouse, clone V9 Cat. MAB3400; Millipore, Billerica, MA, USA (1:200)) and P-Histone3-Alexa 647 (α-rabbit, Cat. 9716S; Cell Signaling, Boston, USA (1:100)). After being rinsed three times with PBS, the cells were incubated with Oregon Green-Alexa647 dye conjugated goat anti-mouse IgG 1:400 (**Life Technologies S.A.**, Alcobendas, Spain) secondary antibodies for 1 hour at room temperature. All cells were counterstained by incubation with 4,6-diamidino-2-phenylindole dihydrochloride (DAPI) from Molecular Probes (Invitrogen, Carlsbad, CA, USA) for 3 min at room temperature followed by washing steps. Samples were mounting using FluorSave Reagent (Calbiochem, Darmstadt, Germany). Signals were visualized by Confocal Microscopy (Leica Microsistemas, Barcelona, Spain). Four different assays were performed and at least 6 different fields per condition and assay were analyzed. 

### 3.9. Statistical Analysis

Statistical comparisons were assessed by Student’s *t*-test. All *P* values were derived from a two-tailed statistical test using the SPSS 11.5 Software. A *P*-value < 0.05 was considered statistically significant. 

## 4. Conclusions

We propose the use of a new strategy that combines the direct implantation of autologous cASCs, obtained and amplified in aseptic conditions, into CLMA proliferative scaffolding structures improved by autologous PRGF addition. This strategy offers the ability to rapidly obtain cASCs in aseptic conditions required for clinical application and reduces the need of massive proliferation and amplification of stem cells by successive culturing. In addition, this proposal avoids further chemotaxis of stem cells into the damaged tissue by *in situ* substitution of the affected tissue with the synthetic scaffold CMLA, which has positive osteoconductive properties [34]. The uniform porosity of CMLA maintains stem cells with homogeneous distribution and allows cASCs to continue with basic biological processes as proliferation. Despite superior biocompatibility of CMLA and the promising results observed after treatment of cASC with PRGF *in vitro*, additional experimentation *in vivo* is required for further translational approaches of the proposed framework to the clinic. Moreover the present data significantly contributes to improve the design of osteoarticular combinatory cell-based therapies where PRGF contributes to enhance stem cell survival in a well adaptable scaffold niche. 
